# Fall Risk in Patient with Behcet’s Disease and Related Factors

**DOI:** 10.5152/eurasianjmed.2022.20368

**Published:** 2022-02-01

**Authors:** Hülya Uzkeser, Ayhan Kul, Yaşar Arslan

**Affiliations:** Department of Physical Medicine and Rehabilitation, Atatürk University Faculty of Medicine, Erzurum, Turkey

**Keywords:** Fall risk, balance, Behçet disease, disease activity

## Abstract

**Objective:** Balance disorders and related falls can cause serious situations that affect the lives of a large number of people and may even be fatal. We have not found any studies about fall risk in Behçet’s disease in the literature before. In this trial, we aimed to investigate the fall risk in patients with Behçet’s disease using an objective computerized technique and evaluated the risk factors for falls in these patients.

**Materials and Methods:** We have included 65 patients with Behçet’s disease and 50 controls in this study. Their vitamin D levels, vitamin B12 levels, and magnesium levels were also determined. The Behçet’s Current Activity Index was used for evaluating disease activity. We used the Falls Efficacy Scale International to evaluate fall efficiency. Tetrax Interactive Balance System was used for posturographic evaluation to objectively determine balance and fall risk.

**Results:** The Behçet’s Current Activity Indices of the patients were 4.17 ± 1.99 (mean ± standard deviation). Fall anamnesis, fall risk assessment, Falls Efficacy Scale International, and visual analog scale levels in the patient group were higher than in the control group. There were also significant differences between the 2 groups for fall anamnesis, fall risk assessment, and visual analog scale values. We found a statistically significant correlation between fall risk with visual analog scale (*r* = 0.437, *P* < .001) and the Behçet’s Current Activity Index (*r* = 0.366, *P* = .003).

**Conclusion:** Our study found that fall risk was higher in patients with Behçet’s disease than in the control group after evaluation by an objective computerized technique. An increase in the risk of falling seems to be related to the activity of the disease.

## Main Points

- The balance was never investigated in Behçet’s disease patients and we did not find any research on this subject. This is the first study about this issue.
- We searched the fall risk in patients with Behçet’s disease by an objective computerized technique.
- The fall risk was found to be higher in patients with Behçet’s disease than the controls.
- This increase in the risk of falling seemed to be related to the activity of the disease. When we compared the correlation of fall risk with clinical parameters, we found a correlation between visual analog scale and Behçet’s Current Activity Index.

## Introduction

Behçet’s disease (BD) was a multisystem vasculitis with recurrent symptoms such as oral and genital ulcerations, skin and ocular involvement, and arthritis. Furthermore, neurological and large vessel involvement can sometimes occur. Causes such as joint diseases, vascular occlusion, neurological problems that we have seen in BD were also the factors that affect balance in humans.^1^ When we looked at the literature, we found that the issue of balance was never investigated in Behçet’s patients and we did not find any research on this subject.

The balance was the totality of the internal and external forces acting on the human body. The main factor in maintaining balance was muscle activity that caused voluntary or involuntary reflex activity.^2^ The skeletal system cannot stand upright against gravity without coordinated muscle activity.^2^

Balance can also be described as a postural adaptation to changes in the center of gravity during rest or activity.^3^ Effective postural responses in this harmony were generated by the integration of proprioceptive, vestibular, and visual data in the central nervous system.^3^ Fall can be defined as an undesirable change of position, which often results in the ground or other lower surface.^4^ In the simplest form, fall can be defined as irreversible displacement.^5^

Individual factors such as age, sex, low or high body weight index, cognitive impairment, previous falls, condition of chronic diseases, and drug use may affect the risk of falls. However, the balance system and postural reflexes may be affected by many different diseases.

Balance disorder and related falls may cause serious situations that may affect the lives of a large number of people and may be fatal. More than one-third of adults who were 65 years and older fall each year.^5^ The problems that may cause serious mortality and morbidity such as hip fractures and head trauma can be seen in 20-30% of the people who fall.^5^ Pre-diagnosis and early treatment are also important for patients with illnesses that may cause loss of balance and increase the risk of falling.

Studies on the risk of falling in some rheumatic diseases such as rheumatoid arthritis and systemic lupus erythematosus have been done previously.^6-7^ Revealing the fall risk and related risk factors in these patients may lead to the prevention of falls and may contribute to patients’ treatment procedures.

We searched the fall risk in patients with BD by an objective computerized technique. However, we also evaluated the risk factors for falls in these patients.

## Materials and Methods

This study was done between November 2018 and September 2019. We received approval from Atatürk University Faculty of Medicine (29.11.2018/07-08). This study was conducted in accordance with the Helsinki Declaration and informed consent was obtained from all participants. Sixty-five patients with BD who were admitted to our outpatient clinic were included in the study. We examined all patients to identify disease activity and involvement. We used the diagnostic criteria of BD which was recommended by the International Studying Group for BD.^8^ We did not include patients who could not cooperate or tolerate fall risk and who had musculoskeletal system diseases or had previous musculoskeletal surgery. Three patients were excluded from the study because of previous musculoskeletal diseases. In the study, 3 patients had additional diseases. One of these patients had hypertension, 1 had asthma, and the other had fibromyalgia. Only 1 patient was using duloxetine because of fibromyalgia. Besides these, all patients were not questioned for fibromyalgia. The control group consisted of 50 healthy persons of the same age and sex as the patients. It was calculated that a sample (62) patients provided a statistical power of 99% for determining a difference in fall risk assessment with an α error of 5%.

We recorded the age, height, weight, and body mass index (BMI) of all participants. The vitamin D levels, vitamin B12 levels, and magnesium levels were also determined.

The Behçet’s Current Activity Index (BCAI) was used for evaluating the disease activity.^9^ This activity form scores (from 0 to 4) the duration of clinical features (oral ulcers, genital ulcers, skin lesions, etc.). We used visual analog scale (VAS) for evaluating pain and the falling story of all participants in the last 12 months was also recorded. Additionally, the fear of falling of the participants was also determined as yes or no.

In this study, we used the Falls Efficacy Scale International (FES-I) to evaluate falls efficiency in patients with BD. This scale is an easy tool to measure the level of anxiety about falling during social and physical activities. The FES-I was built up by the members of the Prevention of Falls Network Europe and its reliability and validity of the Turkish version were done before.^10-11^

We used the Tetrax Interactive Balance System as called static posturography to objectively determine balance and fall risk assessment (Sunlight Medical Ltd., Tel Aviv, Israel). Tetrax static posturography device has a computer and software system that a score was expressed by the device. During the test, the person stands on a standing platform and the pressure sensors detect the displacement patterns in the pressure center. The device determines the risk of falling between 0 and 100 at the end of the procedure. The fall index results divide the fall risk into 3 categories. The low-level fall risk index is 0-36, the medium-level fall risk index is 37-58, and the high-level fall risk is between 59 and 100 points.^12^

### Statistical Analysis

Statistical analysis was performed with Statistical Package for the Social Sciences for Windows version 20.0 (IBM SPSS Corp.; Armonk, NY, USA). Numerical variables are expressed as mean ± standard deviation. Categorical variables are given as n (%). Numerical data were analyzed for normal distribution using the Kolmogorov–Smirnov test. An independent sample *t*-test and Mann–Whitney *U* test were performed to determine statistically significant differences were used which was appropriate. The chi-square test was used to analyze the differences between the groups concerning categorical data. We used Spearman’s correlation test for correlations. *P*-values < .05 were regarded as statistically significant.

## Results

Sixty-two patients with a mean age of 35.39 ± 11.16 (mean ± SD) years constituted the patient’s group. The mean age of the control group was 36.84 ± 11.16 (mean ± SD) years. We did not observe any significant difference between the groups. Demographic parameters such as age, gender, BMI, and levels of vitamin D, B12, and magnesium were also compared, and we did not observe statically significant differences between the groups (Table 1).

Fifty-four patients were using colchicines, 15 patients were using azotyopürin, and 6 patients were using corticosteroids. Only 1 patient was using hydroxyklorokin. The clinical symptoms of the patients were summarized in Table 2. The Behçet’s Current Activity Indices of the patients was 4.17 ± 1.99 (mean ± SD).

Fall anamnesis, fall risk assessment, FES-I, and VAS levels in the patient’s group were higher than the controls. There were also significant differences between the 2 groups according to fall anamnesis, fall risk assessment, and VAS values (Table 3). The patient group has a high fall risk (58-100 is high risk) with the range of 60.68 ± 31.2(mean ± SD) (figure).

We did not find any relationship between fall risk assessment with vitamin D, vitamin B12, and magnesium levels. Also, we investigated the relationship between fall risk with using drugs such as colchium, steroid, and azotyopürin. We did not find a significant difference between drug users and non-users in BD group.

There was no significant difference in terms of fall risk assessment between patients with uveitis and those without uveitis. However, there was a significant difference between patients with arthritis and those without arthritis according to the fall risk assessment (*P* = .012). When we compare the correlation of fall risk with clinical parameters, we find a correlation between VAS and BCAI (Table 4).

We separated the participants according to fall risk assessment categories as low, medium, and high-risk groups and we compared each other. When we divided the participants according to fall risk assessment categories, we found that the participants with a high risk of falling were more in the Behçet group (Table 5). We searched the relationship between all clinical findings with fall risk assessment by making subgroups. We found only statistical significance with arthritis and fall risk (*P* < .05).

## Discussion

Falling, which may lead to significant health outcomes, puts a significant burden on healthcare expenditures worldwide.^6^ Hence, determining the risk of falls and identifying related factors may be useful to prevent falling and treatment management in these patients. We evaluated fall risk and its related factors in patients with BD by using an objective computerized technique in our study. We observed that patients with BD have high fall risk. Also, higher fall risk in these patients seemed to be related to the disease activity. Because of this knowledge, the treatment approach in patients with BD may also be affected.

Behçet’s disease is a systemic inflammatory condition with unknown origin and etiopathogenesis. Some authors consider BD as a neutrophilic vasculitis targeting vasa vasorum, which in turn leads to vessel wall deterioration.^13^ Recently, the authors thought that BD was a complex overlapping disorder, including both autoimmune and autoinflammatory pathogenetic mechanisms.^14^ Turkish dermatologist Hulusi Behçet defined the disease with oral aphthous, genital ulcers, and recurrent uveitis with hypopyon, known as triple-symptom complex.^15^ The other clinical features including musculoskeletal, gastrointestinal, renal, pulmonary, cardiovascular, and cutaneous manifestations were added later.^16^ The ocular, vascular, and neurological involvement were the main causes of morbidity and mortality. In another study, hearing loss was found the fourth most common clinical feature in patients with BD.^17^ We thought that many of the clinical findings in BD may cause balance problems and falls even on their own.

When we search the literature on musculoskeletal issues that can affect balance we find a significant reduction in balance scores and trunk muscle endurance scores has been found in women with fibromyalgia syndrome.^18,19^ It was known that trunk muscles are especially important for maintaining healthy balance during daily life activities, creating proprioceptive data input, protecting spine health, and improving limb function.^20,21^ They were reported that it was important for patients with fibromyalgia syndrome to consider preventive exercise approaches for trunk muscle groups when determining their treatment programs in terms of reducing functional disabilities. Preventive exercise programs may be suggested to patients with BD regarding balance impairment.

The central nervous system is another system that may be affected by BD. The central nervous system was responsible for postural control, stability, and maintaining the balance of the body. The central nervous system worked simultaneously with somatosensorial, vestibular, and visual systems for postural and balance control.^19^ There are anatomical and physiological links between the hearing and balance organs of the body, and hearing impairment affects muscle coordination. Hearing impairment reduces the motor functions by impacting balance, and the decrease in motor functions makes it harder to maintain good posture, postural stabilization, and balance.^22^ The person who has hearing loss has less ability to achieve static balance, to make the body’s dynamic coordination, to move the body’s limbs independently, and to control the speed of movement as compared to normal hearing people.^23^ We also found the fall risk high in this study but we did not examine the auditory and vestibular impairments that could affect the fall in these patients. Because we could not predict these points when we designed the study. This is the limitation of our study. The possible accompanying balance problems with hearing loss and vestibular dysfunction in patients with BD may be analyzed in the future.

We found only statistical significance with arthritis and fall risk BD. When we searched the literature about arthritis and fall risk, we found a study from the United State of America. They investigated in all 50 states, the prevalence of any fall and fall injuries in the past 12 months was significantly higher among adults aged ≥45 years with arthritis compared with those without arthritis.^24^ They evaluated with a self-report. These questions can not assess the type of arthritis, which might affect falls and fall injuries differently. However, an underestimate of self-reported falls can contribute to the results Conversely, the minor falls may report as broad injurious, resulting in an overestimate. We searched the fall risk in patients with BD by an objective computerized technique. There are some trials about rheumatoid arthritis and fall risk. They also find high fall risk but they also did not use an objective computerized technique.^25,26^

We also investigated whether drugs could affect the falling. Many drugs were found associated with impaired balance.^27^ It was determined that the risk of falling in patients with fibromyalgia syndrome aged 50 years or older was associated with drug use.^28^ Especially, antidepressants and antiepileptics such as pregabalin are drugs that can cause serious neurological side effects. In some studies, drug use has been defined as an independent risk factor for balance disorders.^27^ We did not find a study about the relationship between drugs and falls in BD but a few studies about rheumatologic disease indicated that different types of drugs such as glucocorticoids, diuretics, antihypertensives may affect the risk of falling.^6,29^ Contrary to these, we came across another study about steroid use not increasing the risk of falls.^30^ In our study, we investigated the effects of drugs in patient’s groups such as glucocorticoids, colchium, and azotyopürin. We did not find a significant difference between drug users and non-users. But as this study is the first of this issue, it should be further investigated in the future.

In conclusion, the fall risk was found to be higher in patients with BD than the controls, especially evaluated by an objective computerized technique in our study. This increase in the risk of falling seemed to be related to the activity of the disease. New research investigating the possible accompanying balance problems such as vestibular dysfunction, proprioception sensation, muscle strength preservation, postural reflexes, hormone levels, orthostatic hypotension, attention deficit, cognitive symptoms, and vitamin deficiency was needed to investigate BD. These results may contribute to the management of the disease in the future.

## Figures and Tables

**Table 1.** Demographic and Laboratory Parameters of Participants

	Behçet’s Disease (Mean ± SD)	Controls (Mean ± SD)	*P*
Age (year)	35.39 ± 11.01	36.84 ± 11.16	.492^a^
Gender (M/F)	29/33	24/26	.89^b^
BMI	25.38 ± 4.6	26.9 ± 3.9	.058^a^
Vitamin D levels (ng/mL)	18.32 ± 10	20.34 ± 10	.180^c^
Vitamin B12 levels (pg/mL)	303.11 ± 210	298.25 ± 137	.596^c^
Magnesium levels (mg/dL)	1.9 ± 0.17	1.9 ± 0.16	.249^c^

BMI, body mass index; F, female; M, male, SD, standard deviation.

^a^Independent samples *t*-test, ^b^chi-square (2 × 2) independency test, ^c^Mann–Whitney *U* test.

**Table 2.** The Clinical Manifestations of Patients with Behçet’s Disease

	Existence/Non-existence	%
Oral aphthae	44/18	71
Genital aphthae	13/49	21
Uveitis	25/37	40.3
Arthralgia	49/13	79
Arthritis	8/54	12.9
Skin lesions	37/25	59.7
CNS involvement	-	-
Pulmonary embolism	-	-

CNS, central nervous system.

**Table 3.** The Clinical Parameters of Participants

	Behçet’s disease (Mean ± SD)	Controls (Mean ± SD)	*P*
VAS	5.37 ± 3.04	1.9 ± 2.2	<.001^a^
BCAI	4.17 ± 1.99	-	*-*
Fall risk assessment (0-100)	60.68 ± 31.2	32.44 ± 17.7	<.001^a^
FES-I	21.87 ± 9.06	20.7 ± 5.74	*.461* ^a^
Fear of fall (yes/no)	34/28	23/27	*.352* ^b^
Fall anemnesis (yes/no)	52/10	-	*.003*

Statistically significant values are highlighted in italic.

^a^Mann–Whitney *U* test, ^b^chi-square (2 × 2) independency test.

SD, standard deviation; VAS, visual analog scale; BCAI, Behçet’s Current Activity Index; FES-I, Falls Efficacy Scale International.

**Table 4.** The Correlation of Fall Risk Assessment with Clinical Parameters

Fall risk assessment	*r*	*P*
VAS	0.415	<.001^a^
BCAI	0.385	*.002* ^a^

Statistically significant values are highlighted in italic.

^a^Spearman’s correlation test.

VAS, visual analog scale; BCAI, Behçet’s Current Activity Index.

**Table 5.** Fall Assessment with Subgroups

Fall risk assessment	Controls (n) (%)	Behçet’s disease(%)	*P*
Low	35 (63.6)	20 (36.4)	<.001
Medium	12 (48)	13 (52)	
High	3 (9.4)	29 (90.6)
